# Polish Women’s Sexual Strategies in Mate Retention: Initiating Sex, Faking Orgasms, and Performing Oral Sex in Response to Mate Value Discrepancy–Evidence from a Preregistered Study

**DOI:** 10.1007/s10508-026-03423-3

**Published:** 2026-04-24

**Authors:** Natalia Frankowska, Aleksandra Szymkow, Andrzej Galbarczyk

**Affiliations:** 1 Center for Research on Biological Basis of Social Behavior, SWPS University, Warsaw, Poland; 2 Lise Meitner Research Group BirthRites - Cultures of Reproduction, Max Planck Institute for Evolutionary Anthropology, Deutscher Platz 6, 04103 Leipzig, Germany; 3 Department of Environmental Health, Faculty of Health Sciences, Jagiellonian University Medical College, Krakow, Poland

**Keywords:** Oral sex, Fellatio, Faking orgasm, Sex initiation, Mate value, Sexual behavior

## Abstract

Mate retention strategies are behaviors to maintain a partner's commitment and prevent infidelity or relationship dissolution. Individuals generally seek partners with comparable mate value (MV) to reduce the risk of rejection. When a mate value discrepancy (MVD) emerges, the partner with lower MV may adopt strategies to retain the relationship. This study examined whether Polish women with lower MV relative to their male partners more frequently initiated sex, performed fellatio, and faked orgasms, and whether these behaviors were driven by their desire to enhance partner satisfaction. It also investigated whether women’s perceived vulnerability to disease (PVD), their enjoyment of performing fellatio, or their perception of their partner’s enjoyment of receiving fellatio moderated the link between MVD and fellatio frequency, expecting that women less concerned about pathogens or more enjoying the act would show a stronger association. Data from 562 women in committed, sexually active heterosexual relationships showed that higher MVD—where the woman’s MV was lower than her partner’s—indirectly predicted the frequency of initiating sex, performing fellatio, and faking orgasms, through a stronger desire to satisfy the partner sexually. However, PVD, women’s enjoyment of performing fellatio, or their perception of their partner’s enjoyment of receiving fellatio did not significantly moderate the relationship between MVD and fellatio frequency. These results suggest that women use sexual behaviors as benefit-provisioning strategies to strengthen partner commitment, addressing perceived mate value gaps, independent of disease-avoidance concerns or personal and perceived partner enjoyment.

**Supplementary Information:**

The online version contains supplementary material available at 10.1007/s10508-026-03423-3.

## Introduction

Maintaining a stable romantic relationship is beneficial for both men and women. For men, long-term bonds ensure paternity certainty, while women, who invest more biologically in reproduction through pregnancy, childbirth, and lactation, benefit from a committed partner who provides resources, protection, and support, enhancing offspring survival (Fletcher et al., 2015; Quinlan, 2008; Stewart-Williams & Thomas, 2013). A stable relationship increases the likelihood of cooperative parenting, which improves the child’s chances of reaching reproductive age—a key goal in evolutionary terms (Griskevicius et al., 2015). However, maintaining a satisfactory long-term relationship is challenging, due to the fact that both sexes face evolutionary trade-offs between seeking a stable, committed partner, and pursuing alternative mates to maximize reproductive success (Dawson, 1996; Gangestad & Simpson, 2000). As a consequence, people have evolved mate retention strategies designed to mitigate the risks of partner defection and reproductive uncertainty.

### Mate Retention Strategies

Mate retention strategies are behaviors to maintain a partner's commitment and prevent infidelity or relationship dissolution (Buss, 1988). These strategies can be categorized into benefit-provisioning tactics, such as enhancing intimacy or providing resources, and cost-inflicting tactics, like vigilance or mate guarding (Buss & Shackelford, 1997; Miner et al., 2009). Both men and women employ these strategies to a similar extent, though specific tactics may vary. For instance, men tend to lean toward mate guarding, while women toward appearance enhancement (Atari et al., 2017). Cost-inflicting behaviors deter partner infidelity by diminishing the partner’s self-esteem, making them feel unworthy of relationships while benefit-provisioning behaviors prevent it by enhancing the partner’s satisfaction within the relationship (Miner et al., 2009).

An important factor influencing mate retention behaviors in relationships is mate value discrepancy (MVD) (Sela et al., 2017). An individual’s perception of their own mate value—particularly in comparison to their partner’s—appears to affect their motivation to attend more or less to their partner’s needs. When individuals perceive their partner as having higher mate value they may experience insecurity or fear of infidelity, driving them to increase mate retention efforts to maintain the relationship. For instance, a study by Sela et al. (2017) found that women who perceived their partner as having higher mate value reported greater use of benefit-provisioning behaviors, such as enhancing their appearance or offering emotional support, to retain their partner. Similarly, Conroy-Beam et al. (2016) demonstrated that men with higher mate value discrepancy engaged more in cost-inflicting strategies, like mate guarding and jealousy induction (see also Salkicevic et al., 2014).

### Sexual Benefit-Provisioning Mate Retention Strategies

Several studies on mate retention strategies explicitly refer to sexual behavior as a tactic used by both men and women to maintain long-term relationships. For instance, Buss et al. (1988) identified sexual inducements as a distinct category of mate retention tactics. This includes behaviors like initiating sex or using sexual favors to keep a partner interested and committed, with data showing that both genders employ these strategies, though men reported slightly higher use in some contexts. Similarly, Barbaro et al. (2015) linked sexual behavior to infidelity prevention, finding that offering sexual satisfaction was a proactive mate retention strategy reported by both sexes being romantically involved.

Other sexual behaviors, such as performing oral sex or faking orgasms, can also serve as mate retention strategies by reinforcing partner commitment and reducing the risk of infidelity in long-term relationships (Frankowska et al., 2025; Pham et al., 2013; Sela et al., 2015a, 2015b, 2015c).These tactics often function as benefit-provisioning behaviors that increase a partner’s satisfaction or as subtle signals to counter perceived threats from rivals. For instance, Sela et al. (2015b, 2015c) showed that both men and women are more interested in, and spend more time, performing oral sex on their partner as part of a benefit-provisioning strategy to increase their partner’s relationship satisfaction. Also, as Frankowska et al. (2025) evidenced, when mate value discrepancy is taken into account, men who perceive a greater mate value discrepancy favoring their female partners are more motivated to sexually satisfy them, resulting in more frequent cunnilingus. This is consistent with studies indicating that men in long-term, committed relationships, who express strong love for their partners, are more inclined to engage in oral sex with them (Kaestle & Halpern, 2007). Although more studies are needed to explore the specific mechanisms, this suggests that men might engage in cunnilingus to increase their perceived value in their partner's eyes, thereby reducing the mate value discrepancy.

Similarly, in the case of women, not only performing oral sex but also faking orgasms were strategies used by some to boost their partner’s confidence, thereby indirectly strengthening the relationship bond (Sela et al., 2015a). Since men are deeply concerned whether their partner has an orgasm during intercourse (McKibbin et al., 2010; Welling, 2014), women might fake it to maintain their partner’s commitment to the relationship. Indeed, research suggests that pretending to have an orgasm can function as a strategy for mate retention in committed heterosexual relationships and that this behavior is more frequently reported by women (50% of women admit to pretending to have a copulatory orgasm at least once in their lifetime, compared to 25% of men) (Muehlenhard & Shippee, 2010). According to Kaighobadi et al. (2012), women who perceive a higher risk of their partner’s infidelity often engage in faking orgasms, while Younis et al. (2018) reported that 82% of married Egyptian women do so to protect their husband’s self-esteem. Moreover, enhancing a partner's sexual and emotional experience during intercourse is the major explicit reason for faking orgasms reported by women (McCoy et al., 2015). As a consequence, women who fake orgasm frequently have partners with greater self-reported relationship satisfaction compared to women who do not appear to orgasm frequently (Kaighobadi et al., 2012).

Our study aimed to explore whether heterosexual women with a perceived lower mate value than their male partner engage in sex initiation, active oral sex, and faking orgasms more frequently. We hypothesized that a larger mate value gap favoring the male partner would increase the woman's motivation to sexually please him as a mate retention tactic, leading to more frequent attempts at sex initiation, fellatio, and orgasm faking. Therefore, we examined whether the motivation to sexually satisfy the partner mediates the link between mate value discrepancy and the frequency of these behaviors.

Furthermore, for the fellatio frequency, we included a potential moderator of the predicted effects, as research on women's attitudes toward fellatio presents mixed findings. While some women find fellatio satisfying and report positive associations with sexual compatibility and desire in their relationships (Apt et al., 1996), others engage in it repeatedly despite disliking it (Kaestle, 2009). Disgust is the emotion that has been investigated in this context and is evidenced to influence sexual decision-making, though only studied for men (Oaten et al., 2019). The emotion of disgust serves as a pathogen-avoidance mechanism (Curtis et al., 2011) and is a central component of the behavioral immune system, which evolved to shield us from infections (Ackerman et al., 2018; Murray & Schaller, 2016). According to the behavioral immune system theory, avoidant behaviors are highly adaptable and should be more common among those more susceptible to diseases (Ackerman et al., 2018). Research consistently shows that the subjective perception of vulnerability to diseases (PVD) significantly influences pathogen-avoidant behaviors (Duncan et al., 2009; Faulkner et al., 2004). And as engaging actively in oral sex carries a significant risk of exposure to harmful pathogens (Edwards & Carne, 1998), we should expect that vulnerability to diseases would play a role in shaping these sexual decisions. Therefore, in our study, we hypothesized that perceived vulnerability to disease would significantly impact the frequency of active oral sex. Specifically, women with lower disease vulnerability may be more likely to use fellatio as a mate retention strategy, prioritizing the maintenance of committed relationships over pathogen avoidance and health concerns (Tybur et al., 2020).

## Method

The study was pre-registered and the registration is available at https://osf.io/d9zs2/?view_only=f63ab4de1cd84b3da9595edc1513242c. All study materials are available in the Set of Questionnaires in the Supplementary Materials and in the open repository at https://osf.io/ujpwv/overview?view_only=d29cf1cd0d594fc1b740bfdc7bb3a0f5.

### Participants

Participants were recruited through the SONA system (a university participant pool management system) and Facebook advertisements. Female university students who took part via SONA received nominal extra credit in an undergraduate course for their engagement in scientific and social research. The initial sample consisted of 941 participants. In line with our preregistration, the final sample included only adult women who were in a committed, sexually active, heterosexual relationship lasting at least three months and who had completed the full questionnaire. Accordingly, the final sample consisted of 562 Polish women: 477 (84.9%) engaging in exclusively heterosexual sexual activities, and 85 (15.1%) engaging in predominantly heterosexual activities. In accordance with our preregistration, we required a minimum of 485 participants in the final sample. Participants were between 18 and 50 years of age (*M* = 29.65, *SD* = 7.91). Thirty-two (5.7%) were in relationships lasting between 3 and 6 months, 47 (8.4%) between 6 and 12 months, 124 (22.1%) more than 1 year, and 359 (63.9%) more than 3 years.

### Procedure and Measures

Participants were informed that they were taking part in research on sexual behaviors and were asked to complete a web-based survey via Qualtrics (Qualtrics, Provo, UT). Participants first provided demographic information, including their sex, age (in years), primary sexual orientation (exclusively heterosexual, predominantly heterosexual, bisexual, predominantly homosexual, exclusively homosexual, or not sexually active), and the duration of their current relationship (less than 3 months, between 3 and 6 months, between 6 and 12 months, more than 1 year, or more than 3 years). Individuals who meet the inclusion criteria were then asked to report the frequency of three sexual behaviors—initiating sex, performing oral sex, and faking orgasm—during their last 10 sexual encounters, and rated each behavior on a slider scale from 0 to 10. First, participants indicated how many of these encounters were initiated by them, using the question: "Out of your last 10 sexual encounters with your committed partner, how many were initiated by you?" Next, participants reported how many of their last 10 sexual encounters involved orally stimulating their committed male partner, either as part of foreplay or as a substitute for vaginal intercourse, using the question: "Out of your last 10 sexual encounters with your committed partner, how many involved satisfying him orally?" Finally, participants reported how many of their last 10 sexual encounters involved faking orgasm, using the question: "In how many of your last 10 sexual encounters with your committed partner did you fake an orgasm?" We used this approach because it normalizes responses across individuals with varying levels of sexual activity by measuring the proportion of encounters involving the behavior rather than the absolute frequency, which could otherwise be skewed by differences in overall encounter rates.

Additionally, participants were asked about the frequency with which they experienced orgasm and received oral stimulation from their partner during their last 10 sexual encounters. The study's predictor variables included mate value discrepancy, motivation to sexually satisfy the partner, sexual enjoyment, and subjective perceived vulnerability to disease.

**Mate Value Discrepancy.** MVD between female participants and their male committed partners was assessed using the Mate Value Scale (Edlund & Sagarin, 2014). Participants rated their own mate value (MV) and separately evaluated their perception of their partner’s MV. The scale comprised four items measuring perceived MV, capturing how individuals view themselves relative to others in the dating market. Participants were presented with the following instructions: “Many people pay attention to specific traits when choosing a potential romantic partner. Some commonly desired traits include: being socially engaging, age, physical attractiveness, having a sense of humor, being kind and understanding, having high financial/professional status, high intelligence, good health, and whether or not one likes children. Those who possess these traits to a high degree are highly sought after by the opposite sex and are said to have a high mate value. With this in mind, we would like you to assess both your own mate value and the mate value of your romantic partner in the order indicated on the following pages.” After reading the instructions, participants rated themselves and their partners on a 7-point scale (1 = *extremely low* to 7 = *extremely high*) in response to the following four questions: “Overall, how would you rate your level of desirability as a partner?”, “Overall, how would members of the opposite sex rate your level of desirability as a partner?”, “Overall, how do you believe you compare to other people in desirability as a partner?”, and “Overall, how good of a catch are you?” The MVD was calculated by subtracting the average MV of the male partner (Cronbach’s α = .84) from the average MV of female participant (Cronbach’s α = .89). Thus, a higher MVD value indicated a greater difference in the mate value of the male partner relative to the participant's MV in favor of a woman.

**Motivation to Sexually Satisfy the Committed Partner**. To assess participants' overall motivation to sexually satisfy their committed partner, we developed four specific items. Participants rated their agreement with each statement on a 7-point scale (1 = *strongly disagree* to 7 = *strongly agree*). The statements included: "It is most important for me to sexually satisfy my partner," "During sexual intercourse, I engage in additional activities that I know are particularly enjoyable for my partner," "During intimacy, I primarily focus on my own pleasure” (reverse-coded), and "I prioritize my partner's sexual satisfaction over mine during sexual intercourse." The overall motivation to sexually satisfy a committed partner was determined by averaging responses to these four items (Cronbach’s α = .53).

**Perceived Vulnerability to Disease**. Subjective perceptions of susceptibility to disease were assessed using the Perceived Vulnerability to Disease Scale (Duncan et al., 2009). The scale consisted of 15 items rated on a 7-point scale (1 = *strongly disagree* to 7 = *strongly agree*). It included two subscales: Perceived Infectability (e.g., “My immune system protects me from most illnesses that other people get”; PI; Cronbach’s α = .86) and Germ Aversion (e.g., “It really bothers me when people sneeze without covering their mouths”; GA; Cronbach’s α = .72). The items within each subscale were averaged to create two indices, which were analyzed separately.

**Additional Measures.** Participants also answered questions regarding how frequently they received oral stimulation from their partner and experienced orgasm during their last 10 sexual encounters. The questions were: "Out of your last 10 sexual encounters with your committed partner, how many times were you satisfied by him orally?" and “Out of your last 10 sexual encounters with your committed partner, how many times did you experience an orgasm?". Participants rated these frequencies on a slider scale from 0 to 10. Additionally, sexual enjoyment was assessed by asking participants to rate the extent to which they and their romantic partner enjoy specific sexual activities. The four items were: “In your opinion, how much does your partner like being satisfied through oral sex?”, “How much do you like satisfying your partner orally?”, “In your opinion, how much does your partner like being satisfied through vaginal sex?”, and “How much do you enjoy satisfying your partner vaginally?” Participants rated each statement on a 7-point scale (1 = *He definitely doesn’t like/I definitely don’t like* to 7 = *He definitely likes/I definitely like*).

### Statistical Analysis

In line with our preregistered analysis plan, we started by calculating descriptive statistics and conducting Pearson correlation analyses to examine linear relationships between MVD, the frequency of initiating sex, engaging in active oral sex, faking orgasm, and the motivation to sexually satisfy the female romantic partner, using IBM SPSS Statistics 29. Next, we employed the SPSS PROCESS macro, model 4 (Hayes & Rockwood, 2017), to determine whether the relationship between MVD and the frequency of initiating sex, engaging in active oral sex and faking orgasm was mediated by the motivation to sexually satisfy the male romantic partner. These mediations were analyzed separately for each dependent variable. To further explore this, we applied model 7 (Hayes & Rockwood, 2017) to test whether these mediations were moderated by the participant's perceived vulnerability to disease. As recommended by Hayes (2017), we reported unstandardized regression/path coefficients. Additionally, we conducted exploratory analyses that were non-preregistered, which are presented at the end of the Results section.

## Results

**Initial analyses.** Descriptive statistics and correlation coefficients between continuous variables are presented in Table 1. We found that MVD correlated positively with the motivation to sexually satisfy one’s male partner (*r* = .16, *p* < .001), which means that as the mate value discrepancy in favor of the male partner increases, so does the motivation of women to sexually satisfy their partners. However, there was no zero-order correlation between MVD and the frequency of initiating sex (*r* = .07, *p* = .126), performing oral sex (*r* = .04, *p* = .363), and faking orgasm (*r* =−.06, *p* = .176). The motivation to sexually satisfy a partner was positively correlated with the frequency of initiating sex (*r* = .15, *p* < .001), performing oral sex (*r* = .30, *p* < .001), and faking orgasm (*r* = .15, *p* < .001). This indicates that the more motivated a woman was to sexually satisfy her male partner, the more frequently she initiated sex with him, performed oral sex on him, and faked orgasm during sexual encounters. Additionally, the frequency of initiating sex was positively correlated with the frequency of performing oral sex (*r* = .18, *p* < .001), and the frequency of performing oral sex was positively correlated with the frequency of faking orgasm (*r* = .13, *p* = .002). Lastly, the participant's age was negatively related to motivation to sexually satisfy the partner (*r* = -.22, *p* < .001), as well as to the frequency of performing oral sex (*r* = -.10, *p* = .024).

**Table 1:** Zero-order correlations between measured variables

Variables	*M*	*SD*	1	2	3	4	5	6
1. Mate value discrepancy (MVD)	0.65	1.25	–	–				
2. Motivation to satisfy the partner	4.53	1.03	.16***	–				
3. Initiating sex frequency	4.15	2.36	.07	.15***	–			
4. Performing oral sex frequency	4.62	3.09	.04	.30***	.18***	–		
5. Faking orgasm frequency	0.93	2.14	−.06	.15***	.04	.13**	–	
6. Participant’s age (in years)	29.65	7.91	−.06	−.22***	−.05	−.10*	−.07	−

*Note*. Cell entries are zero-order Pearson correlation coefficients (two-tailed), *p < .05, **p < .005, ***p < .001. MVD = subtracted the mate value of a man from the mate value of a woman

Absolute range: Variables 1–2: 1–7; Variables 3–5: 0–10

**The mediating role of motivation to sexually satisfy the partner in the relationship between MVD and the frequency of initiating sex.** To test whether MVD indirectly predicts the frequency of initiating sex through the motivation to sexually satisfy the male romantic partner, we used the SPSS PROCESS macro (Model 4). Given that participants' age and relationship length could influence the frequency of initiating sex, performing fellatio, and faking orgasms we included these variables as covariates in all our analyses.

**Fig. 1:**
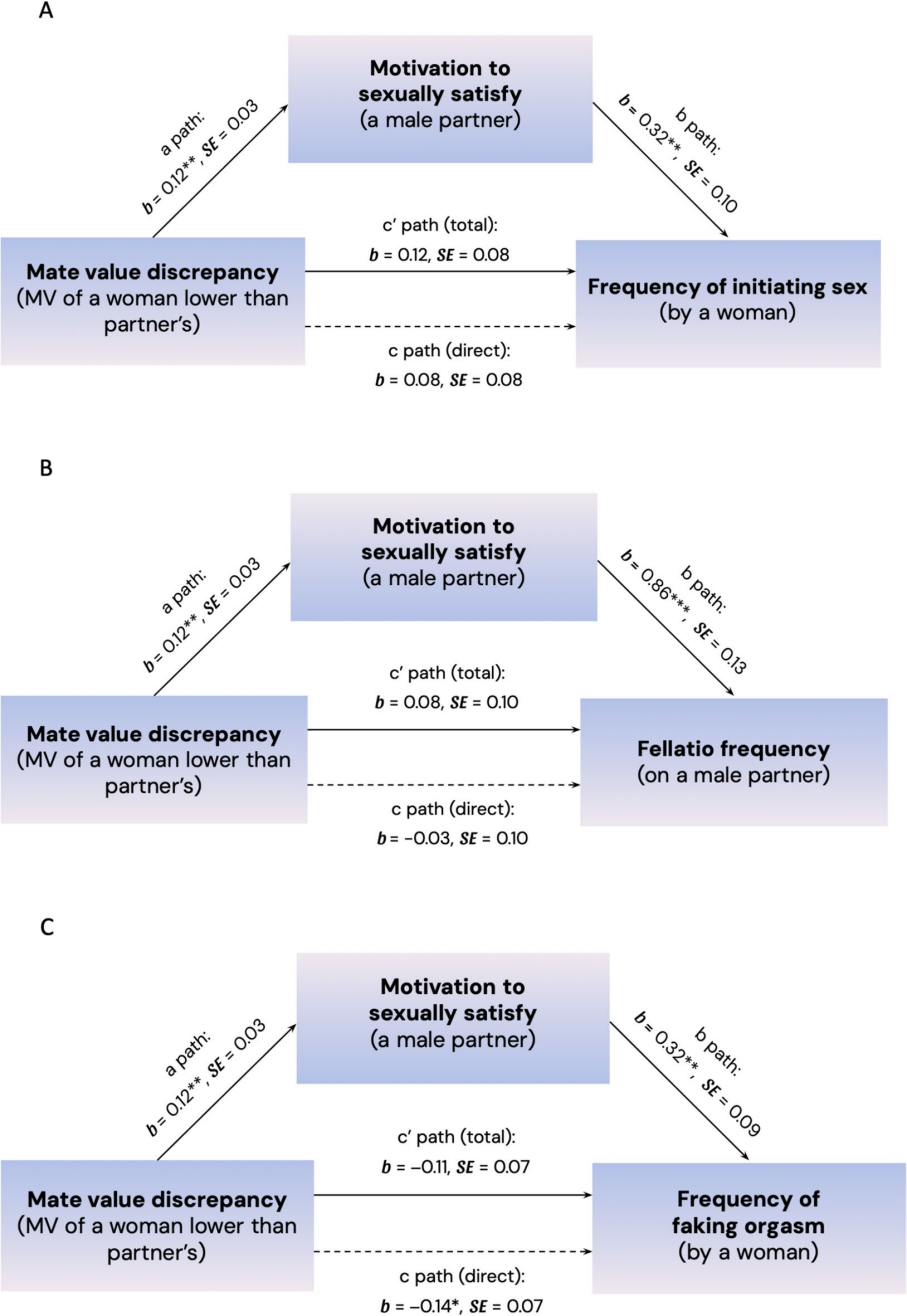
The indirect effect of mate value discrepancy on the frequency of sex initiation (Diagram A), performing fellatio (Diagram B), and faking orgasm (Diagram C) through motivation to sexually satisfy a male partner. Note: *p < .05, **p < .005, ***p < .001. Models included two covariates: participant’s age and relationship length

The results of the mediation analysis (Mate value discrepancy → Motivation to satisfy partner → Frequency of initiating sex) revealed that the predicted indirect effect of the mediation pattern was significant, as indicated by the fact that the 95% CI did not include zero, *b* = 0.039, 95% CI [0.009, 0.078]. This finding is consistent with the hypothesis that the higher the discrepancy between mate value of a woman and her male partner in favor of the partner, the higher her motivation to satisfy the partner, path a: *b* = 0.123, 95% CI [0.057, 0.189], which further predicts the frequency in which she initiates sex, path b: *b* = 0.318, 95% CI [0.122, 0.513]. The direct effect of the MVD on the frequency of initiating sex was not significant, *b* = 0.077, 95% CI [− 0.080, 0.234], nor was the total effect, *b* = 0.116, 95% CI [− 0.040, 0.272]. It means that mate value discrepancy does not predict the frequency of initiating sex by women directly, but indirectly, by affecting the woman’s motivation to satisfy her partner, which further translates into how frequently she initiates sex. The indirect effect diagram is presented in Fig. 1A, and all specific coefficients for direct and indirect paths are presented in Table 2.

**Table 2:** Coefficients of direct and indirect effects of mediation analysis of the relationship between Mate Value Discrepancy and the frequency of initiating sex, performing fellatio, and faking orgasm separately, with one mediator: motivation to sexually satisfy the partner

Motivation to satisfy the partner (M)							Frequency of initiating sex (Y)								Indirect effect coefficients			
	*b*	*SE*	*t*	*p*	*CI* 95%*		*b*	*SE*	*t*	*p*	*CI* 95%*				*b*	*SE*	*CI* 95%*	
					*LL*	*UL*					*LL*		*UL*				*LL*	*UL*
**Direct effects paths**																		
MVD (X)	0.123	0.034	3.665	.000	0.057	0.189	0.078	0.080	0.964	.335	-0.080		0.234					
Motivation to satisfy the partner (M)							0.318	0.100	3.193	.002	0.122		0.513					
Total effect X → Y							0.116	0.80	1.459	.145	-0.040		0.272					
**Indirect effect**																		
MVD → Motivation to satisfy the partner → Frequency of initiating sex															0.039	0.018	0.009	0.078
	Motivation to satisfy the partner (M)						Frequency of performing fellatio (Y)								Indirect effect coefficients			
	*b*	*SE*	*t*	*p*	*CI* 95%*		*b*	*SE*	*t*	*p*	*CI* 95%*				*b*	*SE*	*CI* 95%*	
					*LL*	*UL*					*LL*		*UL*				*LL*	*UL*
**Direct effects paths** MVD (X)	0.123	.034	3.665	.000	0.057	0.189	−0.026	0.101	−0.253	.800	−0.224		0.173					
Motivation to satisfy the partner (M)							0.865	0.126	6.848	.000	0.617		1.113					
Total effect X → Y							0.081	0.104	0.775	.439	-0.124		0.285					
**Indirect effect**																		
MVD → Motivation to satisfy the partner → Frequency of performing fellatio															*0.106*	*0.035*	*0.042*	0.178
	Motivation to satisfy the partner (M)						Frequency of faking orgasm (Y)											
	*b*	*SE*	*t*	*p*	*CI* 95%*		*b*	*SE*	*t*	*p*	*CI* 95%*				*b*	*SE*	*CI* 95%*	
					*LL*	*UL*					*LL*		*UL*				*LL*	*UL*
**Direct effects paths** MVD (X) Motivation to satisfy the partner (M)	0.123	.034	3.665	.000	0.057	0.189	−0.145	0.072	−2.002	.046	−0.287		−0.003					
							0.320	0.090	3.549	.000	0.143		0.498					
Total effect X → Y							−0.105	0.072	−1.460	.145	−0.247		0.036					
**Indirect effect** MVD → Motivation to satisfy the partner → Frequency of performing fellatio															0.039	0.017	0.012	0.077

^*^Note. 95% CI is presented as bias-corrected and accelerated 5,000 bootstrapping. MVD = mate value discrepancy. Covariates: Participant’s age, Relationship length

**The mediating role of motivation to sexually satisfy the partner in the relationship between MVD and the fellatio frequency.** To test whether MVD indirectly predicts the fellatio frequency through the motivation to sexually satisfy the male romantic partner, we used the SPSS PROCESS macro (Model 4; Hayes & Rockwood, 2017). The results of the mediation analysis (Mate value discrepancy → Motivation to satisfy partner → Fellatio frequency) revealed that the predicted indirect effect of the mediation pattern was significant, as indicated by the fact that the 95% CI did not include zero, *b* = 0.106, 95% CI [0.042, 0.178]. This finding is consistent with the hypothesis that the higher the discrepancy between mate value of a woman and her male partner in favor of the partner, the higher her motivation to satisfy the partner, path a: *b* = 0.123, 95% CI [0.057, 0.189], which further predicts the frequency in which she performs fellatio on her male partner, path b: *b* = 0.865, 95% CI [0.617, 1.113]. The direct effect of the MVD on the frequency of performing fellatio was not significant, *b* = -0.026, 95% CI [− 0.224, 0.173], nor was the total effect, *b* = 0.081, 95% CI [− 0.124, 0.285]. It means that mate value discrepancy does not predict the frequency of performing fellatio on male partners directly, but indirectly, by affecting the woman’s motivation to satisfy her partner, which further translates into how frequent she performs fellatio on him. The indirect effect diagram is presented in Fig. 1B, and all specific coefficients for direct and indirect paths are presented in Table 2.

**The mediating role of motivation to sexually satisfy the partner in the relationship between MVD and the frequency of faking orgasms.** To test whether MVD indirectly predicts the frequency of faking orgasms by women through the motivation to sexually satisfy the male romantic partner, we again used the SPSS PROCESS macro (Model 4; Hayes & Rockwood, 2017). The results of the mediation analysis (Mate value discrepancy → Motivation to satisfy partner → Frequency of faking orgasms) revealed that the predicted indirect effect of the mediation pattern was significant, as indicated by the fact that the 95% CI did not include zero, *b* = 0.039, 95% CI [0.012, 0.077]. This finding is consistent with the hypothesis that the higher the discrepancy between the mate value of a woman and her male partner in favor of the partner, the higher her motivation to satisfy the partner, path a: *b* = 0.123, 95% CI [0.057, 0.189], which further predicts the frequency in which she fakes orgasms during sexual encounters, path b: *b* = 0.320, 95% CI [0.143, 0.498]. Although the direct effect of the MVD on the frequency of faking orgasms reached significance, *b* = -0.145, 95% CI [− 0.287, − 0.003], the total effect did not, *b* = -0.105, 95% CI [− 0.247, 0.036]. It suggests that mate value discrepancy does not predict the frequency of orgasms faking directly, but indirectly, by affecting the woman’s motivation to satisfy her partner, which further translates into how frequently she fakes. The indirect effect diagram is presented in Fig. 1C, and all specific coefficients for direct and indirect paths are presented in Table 2.

**The moderating role of perceived vulnerability to disease.** To test whether the above effects are moderated by the participant’s vulnerability to disease, we first calculated zero-order correlations between PVD subscales and performing fellatio frequency. Germ aversion (GA) was negatively, but very weakly, associated with the frequency of performing fellatio (*r* =  − .07, *p* = .048, one-tailed), whereas Perceived Infectability (PI) showed no such relationship (see Supplementary Table 1). This means that the more participants perceived themselves as vulnerable to disease and the more averse they were to germs, the less likely they were to perform fellatio on their male partner. In line with our pre-registered analysis plan, we employed the SPSS PROCESS macro (Model 7; Hayes & Rockwood, 2017) to test whether the indirect relationship between mate value discrepancy (MVD) and the frequency ofperforming fellatio was moderated by PVD. We conducted two separate moderated mediation analyses for an outcome variable: one with GA as the moderator and another with PI as the moderator.

**Germ aversion as a moderator.** Moderated mediation analysis for the frequency of performing fellatio as the outcome variable, MVD as a predictor, motivation to satisfy the male partner as a mediator, and GA as a moderating variable, revealed that the moderation effect was not significant, *b* = -0.021, 95% CI [− 0.078, 0.037]. This indicates that the indirect effect of motivation to satisfy a partner in the link between mate value discrepancy and the frequency of performing fellatio remained significant regardless of female participants’ levels of germ aversion. Detailed findings of GA as a moderator are provided in Supplementary Table 2.

**Perceived infectability as a moderator.** Moderated mediation analysis for the frequency of performing fellatio as the outcome variable, MVD as a predictor, motivation to satisfy the male partner as a mediator, and PI as a moderating variable, revealed that the moderation effect was not significant, *b* = -0.025, 95% CI [− 0.070, 0.029]. It means that the indirect effect of motivation to satisfy a partner in a relationship between mate value discrepancy and the frequency of performing fellatio as the outcome variable held independently of perceived infectability levels. Detailed findings of PI as a moderator are provided in Supplementary Table 3.

**Additional exploratory analyses.** Given the inclusion of additional measures in our study—specifically, the extent to which female participants reported sexual enjoyment, including enjoyment of satisfying their partners through fellatio and vaginal sex, as well as their perceptions of how much their male partners enjoy these activities—we conducted additional exploratory analyses that were not preregistered. First, we calculated zero-order correlations between MVD, women's enjoyment of performing fellatio and vaginal sex, their perceptions of how much their male partners enjoy receiving these activities, and the outcome variables—namely the frequency of sex initiation, performing fellatio, and faking orgasms by female participants. There was a significant correlation between the frequency of performing fellatio and women’s enjoyment of performing it (*r* = .46, *p* < .001), as well as their perception of how much their male partners enjoy receiving it (*r* = .30, *p* < .001). Women's enjoyment of performing fellatio was also significantly correlated with their enjoyment of performing vaginal sex (*r* = .23, *p* < .001), their perception of how much their male partners enjoy receiving vaginal sex (*r* = .21, *p* < .001), and their perception of how much their partners enjoy performing vaginal sex (*r* = .10, *p* = .018). Also, perceived male enjoyment of receiving fellatio was positively correlated with perceived male enjoyment of performing vaginal sex (*r* = .09, *p* = .041). Descriptive statistics and correlation coefficients between these variables are presented in Supplementary Table 4.

As perceptions of whether a partner enjoys receiving fellatio, as well as whether a woman enjoys performing it, can affect the relationship between motivation to satisfy a partner and fellatio frequency, we next examined whether the established indirect effect (Mate Value Discrepancy → Motivation to Satisfy the Partner → Frequency of Performing Fellatio) was moderated by: (1) the female participant’s enjoyment of performing fellatio on her male partner, and (2) the woman’s perception of her male partner’s enjoyment of receiving fellatio. To test this, we first used the SPSS PROCESS macro (Model 14; Hayes & Rockwood, 2017), with female enjoyment of performing fellatio included as a moderator and participant’s age and relationship length entered as covariates. The analysis revealed that the index of moderated mediation was not significant, *b* = 0.004, 95% CI [–0.010, 0.021]. In a second analysis, we again used the SPSS PROCESS macro (Model 14; Hayes & Rockwood, 2017), this time with the female participants’ perception of their male partner’s enjoyment of receiving fellatio included as a moderator and age and relationship length again entered as covariates. The index of moderated mediation was, once more, not significant, *b* = 0.019, 95% CI [–0.001, 0.044]. This means that mate value discrepancy was associated with a higher frequency of performing fellatio through the motivation to satisfy the male partner, regardless of how much the female participants reported enjoying the act or how much they believed their partners enjoyed it. Detailed results are presented in Supplementary Tables 5 and 6.

As participant’s age was significantly correlated with motivation to sexually satisfy the partner and the frequency of performing oral sex, we also tested whether the established indirect effect (Mate Value Discrepancy → Motivation to Satisfy the Partner → Frequency of Performing Fellatio) was moderated by participant’s age. The analysis of Model 7 in SPSS PROCESS macro (Hayes & Rockwood, 2017) indicated that the index of moderated mediation was not significant, *b* = −0.002, 95% CI [–0.011, 0.006]. Thus, the indirect effect stayed true independently of the participant's age. Detailed results are presented in Supplementary Table 7.

## Discussion

The present study provides evidence that mate value discrepancy indirectly affects women’s sexual behaviors in heterosexual relationships through their motivation to sexually satisfy their male partners, supporting the hypothesis rooted in evolutionary psychology that women may employ sexual strategies as a form of mate retention when they perceive their partner as having higher mate value (Conroy-Beam et al., 2016; Frankowska et al., 2025; Miner et al., 2009; Pham et a., 2013; Sela et al., 2015a, 2015b, 2015c). Our findings demonstrate that a greater MVD in favor of the male partner increases a woman’s motivation to satisfy him sexually, which in turn predicts the frequency of initiating sex, performing fellatio, and faking orgasms.

The results of our study align with existing evidence on mate retention strategies, particularly in the context of benefit-provisioning behaviors and responses to perceived mate value discrepancies. These tactics often serve to enhance a partner’s satisfaction or act as subtle signals to counter threats from potential rivals. For example, Sela et al. (2015b, 2015c) demonstrated that both men and women engage more frequently in oral sex as a benefit-provisioning strategy to boost their partner’s relationship satisfaction, a pattern mirrored in our findings on women’s increased frequency of fellatio and sex initiation. Similarly, Frankowska et al. (2025) found that men who perceive a greater mate value discrepancy favoring their female partners are more motivated to sexually satisfy them, leading to more frequent cunnilingus, which parallels our results showing women’s heightened motivation to satisfy their higher mate value male partners through sexual behaviors. Additionally, our findings resonate with Kaestle and Halpern (2007), who noted that men in long-term, committed relationships, who express strong love for their partners, are more likely to engage in oral sex, suggesting that emotional investment and mate retention strategies are closely linked across sexes.

Our study’s findings on faking orgasms align closely with existing research, highlighting its role in relationship dynamics. Similar to Sela et al. (2015a), who indicated that women fake orgasms to boost their partner’s confidence and indirectly strengthen the relationship bond, our results indicate that women’s motivation to sexually satisfy their partner, driven by mate value discrepancy, predicts more frequent faking of orgasms. This is consistent with McKibbin et al. (2010) as well as with Welling (2014), who suggest that men’s concern over their partner’s orgasm prompts women to fake it to maintain partner commitment, a pattern supported by our partial mediation model where mate value discrepancy affects faking orgasms both directly and indirectly. Furthermore, our findings align with prior research showing that women fake orgasms to address perceived infidelity risks (Kaighobadi et al., 2012), to protect their husband's self-esteem (Younis et al., 2018), or to enhance the partner's sexual and emotional experience (McCoy et al., 2015). Additionally, Kaighobadi et al. (2012) found that women who frequently fake orgasms have partners with higher relationship satisfaction, a dynamic that may parallel the increased partner satisfaction implied by our study’s focus on mate retention behaviors. However, while Muehlenhard and Shippee (2010), Mialon (2012), and Wiederman (1997) highlight the higher prevalence of faking orgasms among women compared to men, our study uniquely emphasizes the mediating role of motivation, adding a nuanced layer to understanding this behavior in the context of mate value dynamics.

Our results point to the fact that the impact of MVD on performing fellatio via the motivation to satisfy the partner does not depend on vulnerability to disease, contrary to expectations. Although germ aversion was negatively associated with the frequency of performing fellatio, the correlation was very weak. Importantly, neither germ aversion nor perceived infectability moderated the indirect relation between MVD and oral sex frequency. This effect, although surprising, is consistent with results for men performing cunnilingus on their female partners (Frankowska et al., 2025). In their study, men with lower MV than their partners and higher PVD did not engage in oral sex less frequently to satisfy their partners compared to those with lower PVD. These findings, along with ours, were unexpected, as behavioral immune system theory (Faulkner et al., 2004) suggests that individuals with greater perceived vulnerability to disease are generally more disgust-sensitive and likely to avoid behaviors associated with increased pathogen risks. As suggested by Frankowska et al. (2025), the absence of this effect might stem from the broad nature of the PVD scale employed. The scale (Duncan et al., 2009) evaluates perceived infectability and germ aversion but does not directly measure disgust. Considering the complex nature of disgust, which encompasses different dimensions (e.g., pathogen, moral, and sexual disgust) that serve distinct functional roles (Tybur et al., 2009), the use of a global PVD measure may have obscured more domain-specific mechanisms relevant to sexual behavior. In particular, an interesting path for future research would be to examine the role of sexual disgust in mate retention strategies. Sexual behaviors such as oral sex may simultaneously activate pathogen disgust, due to their association with bodily fluids and infection risk, and moral disgust, insofar as sexual practices are shaped by culturally embedded norms regarding propriety and sexual conduct. While the present study focused on perceived vulnerability to disease, incorporating more targeted measures—such as the sexual and pathogen subscales of the Three Domains of Disgust Scale (TDDS; Tybur et al., 2009) could provide a more fine-grained understanding of how different disgust domains jointly influence intimate behaviors. This approach may help clarify whether individual differences in sexual, pathogen, or moral disgust moderate the relationship between mate value discrepancy and benefit-provisioning behaviors.

Interestingly, our results indicate that MVD plays a significant role in driving the frequency of performing fellatio by women, by increasing their motivation to sexually satisfy their male partner, irrespective of their personal enjoyment of the act. Thus, this motivation appears to operate independently of whether the women themselves find the act enjoyable, highlighting the strength of the underlying psychological mechanism. It suggests that the behavior is more strategically motivated—possibly to maintain the partner’s commitment or satisfaction—rather than being driven by personal pleasure. However, this effect is contrary to what was found by Frankowska et al. (2025) on men, where the mate value discrepancy predicted an increased frequency of cunnilingus but only at moderate and high levels of men’s enjoyment of active oral sex. Men who disliked performing oral sex on their female partners showed no inclination to do so, even when experiencing a significant mate value discrepancy. From an evolutionary perspective, women and men face different reproductive pressures, which can lead to distinct behavioral strategies in relationships. For women, a higher MVD favoring the male partner may trigger greater insecurity about the relationship’s stability, as men with higher mate value are often perceived as having more opportunities for infidelity or mate-switching (Buss & Shackelford, 1997). Our study’s finding that women perform fellatio more frequently regardless of their enjoyment suggests that this behavior may be a strategic mate retention tactic aimed at maintaining the partner’s commitment and satisfaction, potentially reducing the risk of abandonment or infidelity (Kaighobadi et al., 2012). Women, who historically invested more in offspring through pregnancy and childcare, may prioritize behaviors that secure a high-value partner’s investment in the relationship, even at the cost of their own pleasure. Alternatively, the lack of dependence on enjoyment may indicate that women’s motivation is driven more by a perceived need to “compete” with potential rivals and less by personal gratification, reflecting an adaptive response to the perceived threat posed by MVD. For men, on the other hand, these sexual benefit-provisioning mate retention strategies may be less urgency-driven and more pleasure-driven compared to women’s, reflecting a lower evolutionary cost of losing a partner.

In addition to the lack of moderating effect of fellatio enjoyment by women, the indirect effect of MVD on oral sex frequency was also independent of how much women believed their partners enjoyed it. It is plausible that the vast majority of men find receiving oral sex inherently pleasurable, as reflected in studies like Kaestle and Halpern (2007) indicating that men in committed relationships often express strong appreciation for oral sex. Women may be aware of that. Indeed, in our study the mean for perceived partner’s enjoyment of fellatio was very high (*M* = 6.28, *SD* = 1.30, on a scale from 1 to 7), with 66% of women indicating that their partner definitely likes it, 16% reporting that their partner likes it, 8% stating that their partner rather likes it, and only 5.9% indicating any level of dislike. As a consequence, if most men indeed enjoy fellatio, women may not need to heavily weigh their partner’s perceived enjoyment when deciding to engage in this behavior as a mate retention tactic. Future research should explore whether men’s actual enjoyment, rather than women’s perceptions, moderates these patterns.

While this study provides insights into the role of MVD in driving women’s sexual behaviors as mate retention strategies, several limitations must be acknowledged. First, the study relied on self-reported data regarding sensitive topics which may lead to underreporting or overreporting certain behaviors to align with perceived social norms. Indeed, Polish women often exhibit conservative attitudes toward sex, influenced by cultural and religious factors (Gescinska, 2010; Treas et al., 2014). Poland’s strong Catholic heritage, with over 90% of the population identifying as Catholic (Romański-Cebula, 2023), emphasizes traditional gender roles and sexual restraint, particularly for women. Although oral sex is not generally considered taboo in Poland and is becoming increasingly common (Izdebski, 2020), this is not the case in other cultures (Keating, 1988; Pakpahan et al., 2022). Thus, the moderating role of cultural factors should be investigated in future studies. Similarly, disgust sensitivity warrants further exploration, as varying levels of pathogen burden might influence perceived vulnerability to disease (Miłkowska et al., 2021; Szymkow et al., 2021; Tybur et al., 2016), potentially affecting oral sex practices. At the same time, it should be highlighted that although the study revealed several significant correlations supporting the hypothesized relationships, a notable limitation is that many of these associations were relatively weak in magnitude (typically in the small to small-to-moderate range). This suggests that the examined variables explain only a limited portion of the variance in the outcomes, potentially reducing the practical significance of the findings and highlighting the influence of unmeasured factors.

Second, it must be emphasized that the reliability of the motivation to satisfy the partner scale (Cronbach’s α = .53) was substantially lower than desirable in the present sample. The low internal consistency observed here may have attenuated the mediated effects involving this variable. Replication with a more reliable measure of this construct is needed before firm conclusions can be drawn. Also, the use of the Perceived Vulnerability to Disease scale (Duncan et al., 2009) to assess the moderating role of disgust sensitivity in the indirect relation between MVD and fellatio frequency was a limitation, as it did not directly measure sexual aspect of disgust, which may be more relevant to the behaviors studied. Third, the study did not account for cultural or individual differences in relationship dynamics, such as sociosexuality, religiosity, and personality traits, which could affect how MVD and motivation translate into sexual behaviors across diverse populations. For instance, Sela et al. (2015c) demonstrated that women who score higher in conscientiousness and agreeableness exhibit increased interest in and dedicate more time to performing fellatio on their partners. Finally, while we examined women’s perceptions of their partner’s enjoyment of fellatio, we did not directly measure the male partners’ actual enjoyment, which could provide a more accurate picture of how partner preferences influence these behaviors.

In conclusion, this study emphasizes the role of mate value discrepancy in shaping women’s sexual behaviors as mate retention strategies, indicating that a greater mate value discrepancy in favor of the male partner drives increased motivation to sexually satisfy him, thereby indirectly predicting the frequency of initiating sex, performing fellatio, and faking orgasms. From an evolutionary perspective, these findings highlight how women may strategically employ sexual behaviors to secure a high-value partner’s commitment, reflecting adaptive responses to perceived relationship threats like infidelity or mate-switching (Buss & Shackelford, 1997). By demonstrating the independence of these effects from factors like vulnerability to disease or perceived partner enjoyment, this study emphasizes the robustness of evolutionary mechanisms in modern relationship dynamics, offering valuable insights for understanding sexual strategies and their implications for relationship stability.

## Supplementary Information

Below is the link to the electronic supplementary material.

Supplementary file1 (DOCX 46 KB)

## Data Availability

Dataset and all study materials are available in the Set of Questionnaires in the Supplementary Materials and in the open repository at https://osf.io/ujpwv/overview?view_only=d29cf1cd0d594fc1b740bfdc7bb3a0f5.
