# Supplementary material for: Polish Women’s Sexual Strategies in Mate Retention: Initiating Sex, Faking Orgasms, and Performing Oral Sex in Response to Mate Value Discrepancy–Evidence from a Preregistered Study
Source: Arch Sex Behav. 2026 Apr 24;55(3):1179–91. doi: 10.1007/s10508-026-03423-3 (PMC13194317; doi:10.1007/s10508-026-03423-3)
Supplement: Supplementary file 1 — Supplementary file1 (DOCX 46 KB) [file 10508_2026_3423_MOESM1_ESM.docx]

**Supplementary Materials**

To accompany the manuscript “Women’s Sexual Strategies in Mate Retention: Initiating Sex, Faking Orgasms, and Performing Oral Sex in Response to Mate Value Discrepancy

– Evidence from a Preregistered Study”

## **Set of Questionnaires**

**Mate value discrepancy (The Mate Value Scale^21^)**

Many people look at specific characteristics in choosing their potential marriage partners. Some common desirable traits include: being socially exciting, age, being physically attractive, having a good sense of humor, being kind and understanding, having good financial/professional status, being of high intelligence, being in good health, and liking children:

Overall, how would you rate your level of desirability as a partner on the following scale?

1 2 3 4 5 6 7

Extremely Extremely

undesirable desirable

Overall, how would members of the opposite sex rate your level of desirability as a partner

on the following scale?

1 2 3 4 5 6 7

Extremely Extremely

undesirable desirable

Overall, how do you believe you compare to other people in desirability as a partner on the following scale?

1 2 3 4 5 6 7

Very much Lower Slightly Average Slightly Higher Very much

lower than than lower than higher than than higher than

average average average average average average

Overall, how good of a catch are you?

1 2 3 4 5 6 7

Very bad Bad Somewhat Average Somewhat Good Very good

catch catch bad of a catch good of a catch catch

catch catch

### **Motivation to sexually satisfy the committed partner**

For each statement about sexual encounters with your committed partner, please check the box that best represents your level of agreement on a scale from 1 = strongly disagree to 7 = strongly agree:

### 1. It is most important for me to sexually satisfy my partner.

### 2. During a sexual encounter, I engage in additional activities that I know are particularly enjoyable for my partner.

### 3. During intimacy, I primarily focus on my own pleasure. R

### 4. I prioritize my partner's sexual satisfaction over mine during a sexual encounter.

### **Perceived vulnerability to disease (PVD scale^45^)**

For each statement considering your thoughts about how diseases are spreading - please check the box corresponding to the answer that best represents your level of agreement with each of them on a scale from 1 = strongly disagree to 7 = strongly agree:

1. It really bothers me when people sneeze without covering their mouths.
2. If an illness is 'going around', I will get it.
3. I am comfortable sharing a water bottle with a friend. R
4. I don't like to write with a pencil someone else has obviously chewed on.
5. My past experiences make me believe I am not likely to get sick even when my friends are sick. R
6. I have a history of susceptibility to infectious diseases.
7. I prefer to wash my hands pretty soon after shaking someone's hand.
8. In general, I am very susceptible to colds, flu, and other infectious diseases.
9. I dislike wearing used clothes because you don't know what the past person who wore it was like.
10. I am more likely than the people around me to catch an infectious disease.
11. My hands do not feel dirty after touching money. R
12. I am unlikely to catch a cold, flu, or other illness, even if it is going around. R
13. It does not make me anxious to be around sick people. R
14. My immune system protects me from most illnesses that other people get. R
15. I avoid using public telephones because of the risk that I may catch something from the previous user.

**Tables**

**Supplementary Table 1**

*Zero-order correlations among PVD subscales (Germ Aversion and Perceived Infectability) and Performing Oral Sex Frequency*

| **Variables** | ***M*** | ***SD*** | 1 | 2 | 3 |
| --- | --- | --- | --- | --- | --- |
| 1. Germ aversion (GA) | 3.92 | .97 | - |  |  |
| 2. Perceived infectability (PI) | 3.95 | 1.12 | .34*** | - |  |
| 3. Performing oral sex frequency | 4.62 | 3.09 | -.07* | -.02 | - |

*Note*. Cell entries are zero-order Pearson correlation coefficients (one-tailed), **p* < .05, ***p* < .005, ****p* < .001.

**Supplementary Table 2**

*Moderated Mediation Model 7: Indirect effect of Mate Value Discrepancy (IV) on Fellatio Frequency (DV) through Motivation to Satisfy the Partner (M) moderated by Germ Aversion (Mo), Covariates: participant’s age, relationship length, N = 562*

|  | **Mediator variable model (DV = Motivation)** | | | | |
| --- | --- | --- | --- | --- | --- |
| **Predictors** | *b* | *SE* | *t*-Value | *p*-Value |  |
| Mate value discrepancy (MVD) | 0.225 | 0.137 | 1.649 | .100 |  |
| Germ Aversion (GA) | -0.011 | 0.049 | -0.229 | .819 |  |
| MVD x GA | -0.024 | 0.032 | -0.756 | .450 |  |
|  | **Dependent variable model (DV = Fellatio Frequency)** | | | | |
| **Predictors** | *b* | *SE* | *t*-Value | *p*-Value |  |
| Mate value discrepancy (MVD) | -0.026 | 0.101 | -0.253 | .800 |  |
| Motivation to satisfy the partner | 0.865 | 0.126 | 6.848 | .000 |  |
|  | **Conditional Indirect Effect at GA +/- 1 SD** | | | | |
| **Moderator** | Moderator value | *b* | Boot *SE* | Boot 95% CI* |  |
| Germ Aversion (GA) | (-1 SD) 3.95 | 0.133 | 0.048 | [.044, .234] |  |
|  | (Mean) 3.92 | 0.112 | 0.036 | [.049, .191] |  |
|  | (+1 SD) 4.90 | 0.092 | 0.043 | [.016, .184] |  |
| **Index of moderated mediation** |  | -0.021 | 0.029 | [-.078, .036] |  |

*Note*. ** 95% CI is presented as bias-corrected and accelerated 5,000 bootstrapping.*

**Supplementary Table 3**

*Moderated Mediation Model 7: Indirect effect of Mate Value Discrepancy (IV) on Fellatio Frequency (DV) through Motivation to Satisfy the Partner (M) moderated by Perceived Infectability (Mo), Covariates: participant’s age, relationship length, N = 562*

|  | **Mediator variable model (DV = Motivation)** | | | | |
| --- | --- | --- | --- | --- | --- |
| **Predictors** | *b* | *SE* | *t*-Value | *p*-Value |  |
| Mate value discrepancy (MVD) | 0.240 | 0.119 | 2.019 | .044 |  |
| Perceived Infectability (PI) | 0.022 | 0.044 | 0.502 | .616 |  |
| MVD x PI | -0.029 | 0.028 | -1.026 | .305 |  |
|  | **Dependent variable model (DV = Fellatio Frequency)** | | | | |
| **Predictors** | *b* | *SE* | *t*-Value | *p*-Value |  |
| Mate value discrepancy (MVD) | -0.026 | 0.101 | -0.253 | .800 |  |
| Motivation to satisfy the partner | 0.865 | 0.126 | 6.848 | .000 |  |
|  | **Conditional Indirect Effect at PI +/- 1 SD** | | | | |
| **Moderator** | Moderator value | *b* | Boot *SE* | Boot 95% CI* |  |
| Perceived Infectability (PI) | (-1 SD) 2.83 | 0.138 | 0.047 | [.048, .232] |  |
|  | (Mean) 3.95 | 0.110 | 0.035 | [.047, .186] |  |
|  | (+1 SD) 5.06 | 0.083 | 0.043 | [.010, .177] |  |
| **Index of moderated mediation** |  | -0.025 | 0.025 | [-.069, .028] |  |

*Note*. ** 95% CI is presented as bias-corrected and accelerated 5,000 bootstrapping.*

**Supplementary Table 4**

*Zero-order correlations among independent and dependent variables*

| **Variables** | ***M*** | ***SD*** | 1 | 2 | 3 | 4 | 5 | 6 |
| --- | --- | --- | --- | --- | --- | --- | --- | --- |
| 1. Mate value discrepancy (MVD) | .65 | 1.25 | - |  |  |  |  |  |
| 2. Female’s enjoyment of performing fellatio | 5.02 | 1.76 | .08 | - |  |  |  |  |
| 3. Female’s enjoyment of performing vaginal sex | 6.34 | 1.14 | .05 | .23*** | - |  |  |  |
| 4. Male’s enjoyment of receiving fellatio | 6.28 | 1.30 | -.03 | .21*** | .02 | - |  |  |
| 5. Male’s enjoyment of performing vaginal sex | 6.51 | .95 | -.02 | .10** | .29*** | .09* | - |  |
| 6. Fellatio frequency | 4.11 | 3.28 | .04 | .46*** | .08 | .30*** | -.05 | - |

*Note*. Cell entries are zero-order Pearson correlation coefficients (two-tailed), **p* < .05, ***p* < .005, ****p* < .001.

**Supplementary Table 5**

*Moderated Mediation Model 14: Indirect effect of Mate Value Discrepancy (IV) on Fellatio Frequency (DV) through Motivation to Satisfy the Partner (M) moderated by the female participant’s Enjoyment of Performing Fellatio on her male partner, (Mo), Covariates: participant’s age, relationship length, N = 562.*

|  | **Mediator variable model (DV = Motivation)** | | | | |
| --- | --- | --- | --- | --- | --- |
| **Predictors** | *b* | *SE* | *t*-Value | *p*-Value |  |
| Mate value discrepancy (MVD) | 0.122 | 0.034 | 3.665 | .000 |  |
|  | **Dependent variable model (DV = Fellatio Frequency)** | | | | |
| **Predictors** | *b* | *SE* | *t*-Value | *p*-Value |  |
| Mate value discrepancy (MVD) | 0.322 | 0.092 | -0.604 | .546 |  |
| Motivation to satisfy the partner | 0.322 | 0.320 | 1.006 | .315 |  |
| Women’s enjoyment of performing fellatio | 0.576 | 0.272 | 2.117 | .035 |  |
| MVD x Women’s enjoyment of performing fellatio | 0.034 | 0.062 | 0.546 | .586 |  |
|  | **Conditional Indirect Effect at Moderator +/- 1 SD** | | | | |
| **Moderator** | Moderator value | *b* | Boot *SE* | Boot 95% CI* |  |
| Women’s enjoyment of performing fellatio | (-1 SD) 3.26 | 0.053 | 0.025 | [.012, .109] |  |
|  | (Mean) 5.02 | 0.060 | 0.025 | [.019, .116] |  |
|  | (+1 SD) 6.77 | 0.068 | 0.031 | [.017, .138] |  |
| **Index of moderated mediation** |  | 0.004 | 0.008 | [-.011, .021] |  |

*Note*. ** 95% CI is presented as bias-corrected and accelerated 5,000 bootstrapping.*

**Supplementary Table 6**

*Moderated Mediation Model 14: Indirect effect of Mate Value Discrepancy (IV) on Fellatio Frequency (DV) through Motivation to Satisfy the Partner (M) moderated by the female participants’ perception of their partner's enjoyment of receiving fellatio, (Mo), Covariates: participant’s age, relationship length, N = 562*

|  | **Mediator variable model (DV = Motivation)** | | | | |
| --- | --- | --- | --- | --- | --- |
| **Predictors** | *b* | *SE* | *t*-Value | *p*-Value |  |
| Mate value discrepancy (MVD) | 0.123 | 0.034 | 3.665 | .000 |  |
|  | **Dependent variable model (DV = Fellatio Frequency)** | | | | |
| **Predictors** | *b* | *SE* | *t*-Value | *p*-Value |  |
| Mate value discrepancy (MVD) | 0.002 | 0.097 | 0.023 | .982 |  |
| Motivation to satisfy the partner | -0.204 | 0.564 | -0.364 | .718 |  |
| Women's perception of their partner's enjoyment of receiving fellatio | 0.014 | 0.382 | 0.037 | .971 |  |
| MVD x Women's perception of their partner's enjoyment of receiving fellatio | 0.151 | 0.088 | 1.707 | .089 |  |
|  | **Conditional Indirect Effect at Moderator +/- 1 SD** | | | | |
| **Moderator** | Moderator value | *b* | Boot *SE* | Boot 95% CI* |  |
| Women's perception of their partner's enjoyment of receiving fellatio | (-1 SD) 4.98 | 0.546 | 0.166 | [.221, .871] |  |
|  | (Mean) 6.28 | 0.742 | 0.123 | [.501, .982] |  |
|  | (+1 SD) 7.00 | 0.850 | 0.140 | [.576, 1.124] |  |
| **Index of moderated mediation** |  | 0.019 | 0.011 | [-.001, .043] |  |

*Note*. ** 95% CI is presented as bias-corrected and accelerated 5,000 bootstrapping.*

**Supplementary Table 7**

*Moderated Mediation Model 7: Indirect effect of Mate Value Discrepancy (IV) on Fellatio Frequency (DV) through Motivation to Satisfy the Partner (M) moderated by Participant’s Age (Mo), Covariate: relationship length, N = 562*

|  | **Mediator variable model (DV = Motivation)** | | | | |  |
| --- | --- | --- | --- | --- | --- | --- |
| **Predictors** | *b* | *SE* | *t*-Value | *p*-Value |  | |
| Mate value discrepancy (MVD) | 0.123 | 0.034 | 3.665 | .000 |  | |
| Participant’s age | -0.023 | 0.006 | -3.707 | .000 |  | |
| MVD x Participant’s age | -0.003 | 0.005 | -0.565 | .573 |  | |
|  | **Dependent variable model (DV = Fellatio Frequency)** | | | | |  |
| **Predictors** | *b* | *SE* | *t*-Value | *p*-Value |  | |
| Mate value discrepancy (MVD) | -0.025 | 0.101 | -0.243 | .808 |  | |
| Motivation to satisfy the partner | 0.877 | 0.124 | 7.071 | .000 |  | |
|  | **Conditional Indirect Effect at Moderator +/- 1 SD** | | | | |  |
| **Moderator** | Moderator value | *b* | Boot *SE* | Boot 95% CI* |  | |
| Participant’s age | (-1 SD) 22.00 | 0.123 | 0.048 | [.035, .222] |  | |
|  | (Mean) 28.00 | 0.109 | 0.036 | [.043, .184] |  | |
|  | (+1 SD) 38.00 | 0.087 | 0.050 | [-.008, .188] |  | |
| **Index of moderated mediation** |  | -0.002 | 0.004 | [-.011, .006] |  | |

*Note*. ** 95% CI is presented as bias-corrected and accelerated 5,000 bootstrapping.*
